# A new approach for detecting low-level mutations in next-generation sequence data

**DOI:** 10.1186/gb-2012-13-5-r34

**Published:** 2012-05-23

**Authors:** Mingkun Li, Mark Stoneking

**Affiliations:** 1 Department of Evolutionary Genetics, Max Planck Institute for Evolutionary Anthropology, D04103, Leipzig, Germany

## Abstract

We propose a new method that incorporates population re-sequencing data, distribution of reads, and strand bias in detecting low-level mutations. The method can accurately identify low-level mutations down to a level of 2.3%, with an average coverage of 500×, and with a false discovery rate of less than 1%. In addition, we also discuss other problems in detecting low-level mutations, including chimeric reads and sample cross-contamination, and provide possible solutions to them.

## Background

Next-generation sequencing (NGS) is now widely used in biological and medical studies. Most re-sequencing studies have the goal of identifying homozygous or heterozygous mutations in diploid genomes (that is, mutations present at 50% or 100% frequency in sequence reads), and use this information to study genome evolution, infer population history, or identify causal genes/mutations in disease-association studies [1,2]. However, some applications require the identification of low-level mutations (LLMs) that are present at frequencies well below 50% within the population of molecules that is typically sequenced in an NGS study; examples include heteroplasmic mutations in mitochondrial DNA (mtDNA) genomes [3], somatic mutations in tumors [4], or mutations in pooled DNA samples [5].

Challenges in detecting true LLMs come from sequencing error, library contamination, PCR artifacts, and so on. Sequencing error is the most common problem; for instance, the Illumina Genome Analyzer, which is one of the most popular NGS platforms, has an average error rate of 0.01 [6]. Moreover, sequencing error is unevenly distributed along the genome and may be influenced by the sequence context, position on the read, and molecule structure, resulting in sequencing error 'hot spots' where the error rate can be ten-fold greater (or more) than the genome average [3,7-10]. Unfortunately, those features resulting in sequencing error hot spots have not been fully characterized, thus making it difficult to distinguish sequencing errors from true LLMs [10].

Detecting 'true' mutations involves genotype estimation (that is, the mutation frequency is expected to be 0%, 50%, or 100% for diploid data), and methods exist to provide accurate inference at a coverage of around 20× [2,11]. By contrast, even though much higher sequencing depth is typically obtained for NGS studies designed to detect LLMs (often ≥1,000×), the challenge remains to distinguish LLMs from sequencing errors [12]. Recently, several attempts have been made, either by modifying the sequencing library protocol [13,14], or using control data or population data to identify the erroneous base call [15-20]. However, most of these computational methods require some parameters to be set, such as the expected haplotype number, one or more threshold(s) to define the real LLM, and/or which part of the reads to use; hence, these are subjective and can be difficult to implement.

**Figure 1:**
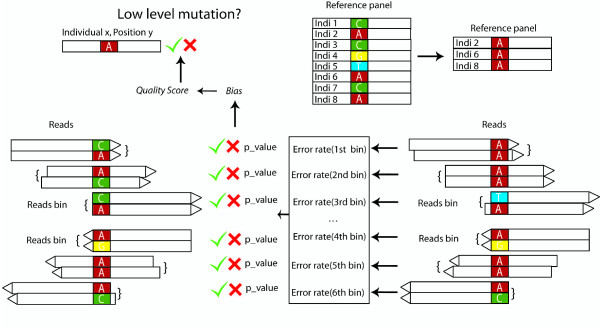
**Workflow of the pipeline**. For each position in the target region, samples having the same consensus nucleotide are used as reference samples. Reads are categorized into different bins according to their positions and orientation when mapped to the reference genome; reads mapped to the questioned sample are also assigned to the same set of bins. By comparing the minor allele count and expected error count, bins are divide into two categories: bins with minor allele count equal or less than the expected error (derived from the reference panel; denoted by a green check mark), and bins with minor allele count greater than the expected error (red cross). Different methods (Poisson, Fisher exact, Empirical) were used to calculate the *P*-value, which represents the deviation of the observation from expectation under the error model. The *P*-values are then used to calculate the bias statistic, which is further converted to a Phred-like quality score to represent the uncertainty concerning the minor allele at this position.

We analyzed PhiX 174 and mtDNA sequencing data, and identified sequencing error hot spots, even under a stringent quality filter, that cannot be explained by the sequence context. However, we find that sequencing error is strand-dependent, position-dependent, and the same sequencing error hot spot repeatedly showed up among different individuals. Based on these features, we have developed a new approach to distinguish LLMs from sequencing errors, which makes use of population re-sequencing data to estimate the sequencing error profile, and gives an understandable Phred-like quality score to present the reliability of the minor allele at each position. The workflow for the method is outlined in Figure 1. This approach thus provides the investigator the flexibility of applying different discovery strategies, that is, a higher false positive rate with a lower false negative rate, or a lower false positive rate with a higher false negative rate. We apply our approach to simulated data, artificial mixtures, and a dataset of complete mtDNA genome sequences, and we demonstrate the method can accurately identify LLMs down to a level of 2% (with an average coverage of 500×) with a low false discovery rate (< 1%). Our method outperformed other existing software in detecting LLMs, especially at positions where the error allele count is low.

## Results

### Sequencing error along the genome

Under the quality filter we used (details in Materials and methods), the average genome-wide error rate (minor allele frequency) is 0.0009 for the PhiX174 genome, and 0.00167 for the mtDNA genome. This difference could be caused by heteroplasmy and/or alignment problems with mtDNA. Generally, the sequencing error rate fluctuated along the genome with some striking peaks (drops when converted to Phred quality score; Figure S1 in Additional file 1), with two peaks in the PhiX174 genome corresponding to true 'polymorphic' positions (mixture of two different alleles) in this PhiX174 strain (positions 1401,1644). Outliers in the mtDNA genome are positions 309 to 311, 514, and 3,106 to 3,107, which are either caused by alignment problems or true length heteroplasmies.

**Figure 2:**
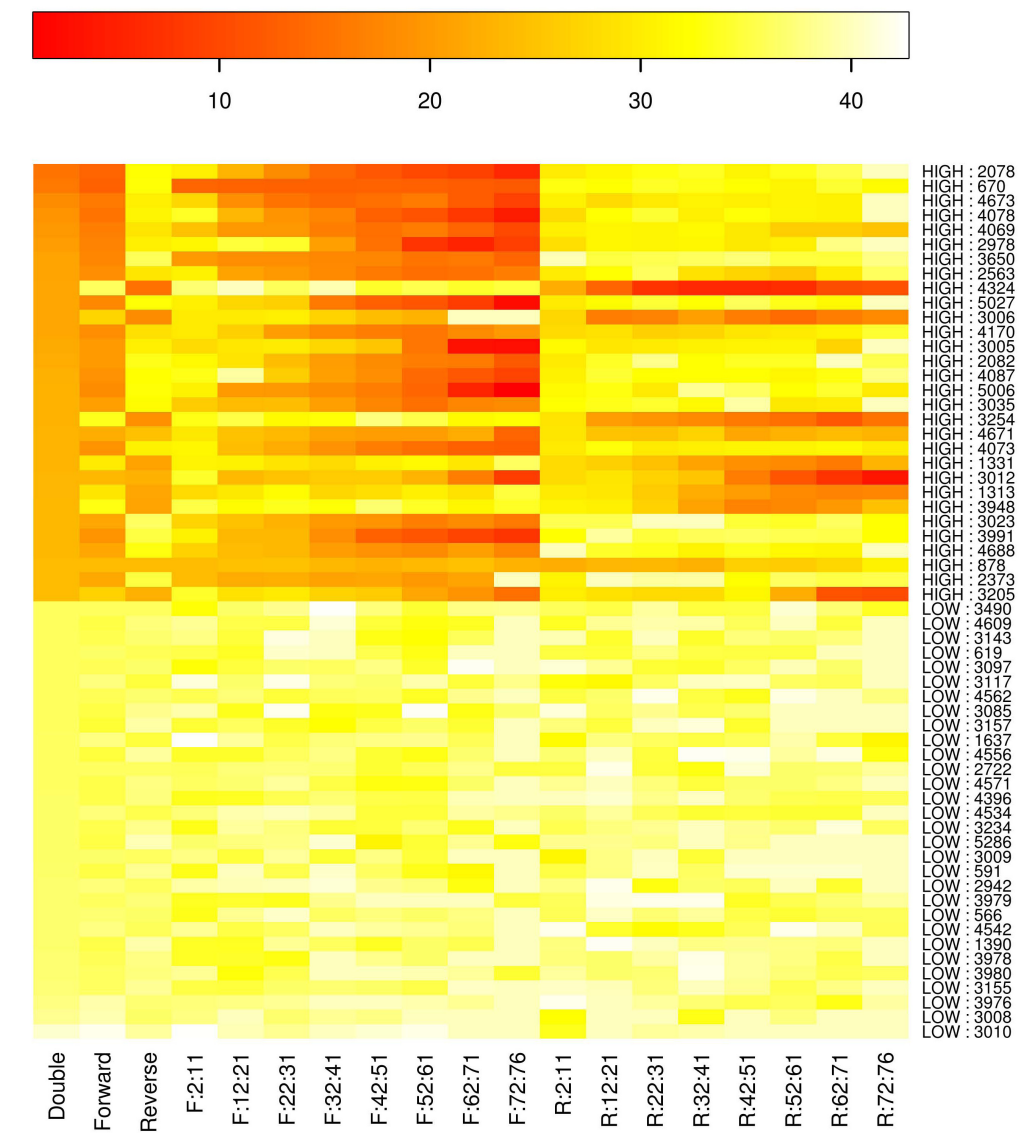
**Sequencing error rate for different parts of the read**. Along the y-axis, the first 30 positions have the highest error rate on the PhiX174 genome, and the last 30 positions have the lowest error rate. The x-axis indicates the strand (F, forward; R, reverse) and read bins (positions 2 to 11, 12 to 21, 22 to 31, 32 to 41, 42 to 51, 52 to 61, 62 to 71, and 72 to 76).

Normally, positions with the highest sequencing error rate cause the most problems in distinguishing LLMs; hence, we retrieved the 30 positions with the highest error rate on the PhiX174 genome to visualize the distribution of error rates along reads, as well as 30 positions with the lowest error rate for comparison (Figure 2). First, an obvious error rate difference was observed between the two strands: positions with high error rates were mostly dominated by reads mapped to one specific strand whereas reads mapped to the other strand showed a normal error rate. Additionally, the error rate also varied among different parts of the reads; although error rate tended to increase when closer to the end, the trend is much weaker on the reads from the low-error strand (Figure 2).

We used WebLogo [21] to identify possible conserved motifs preceding the sequencing error hot spots (Figure S2A in Additional file 1). Although 'GGT' was found to be the most abundant motif preceding sequencing error hot spots on both strands, there is substantial variation in sequencing error rates at positions following this motif in the PhiX174 genome (Figure S2B in Additional file 1). Therefore, this motif alone is insufficient to trigger a higher error rate. This is also true for the 'AAA' motif associated with sequencing error cold spots (Figure S2C, D in Additional file 1) and all the other 3-bp motifs (Figure S3 in Additional file 1), as the error rate following the same motif shows ten-fold variation. Thus, only a small fraction of the variation in sequencing error variation can be explained by 3-bp motifs consisting of the 2-bp context and the nucleotide itself.

**Figure 3:**
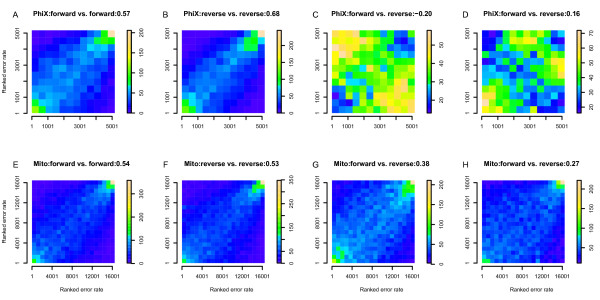
**Sequencing error rate correlation between different subsets of the data**. Sequencing errors are ranked from lowest to highest along each axis. The color in each bin represents the number of positions having the corresponding error rates in the two subsets. **(a-d) **Phix174 data. **(e-h) **mtDNA data. Correlation coefficients are shown at the end of the title of each plot.

In our previous study, the same error hot spots were repeatedly observed in different individuals [3], suggesting that the sequencing error rate could be predicted using population data. To test this hypotheses, PhiX174 data were divided into two subsets according to different sequencing runs, while the mtDNA data were also divided into two subsets according to different read lengths (36 bp versus 76 bp). Since the estimated sequencing error rate for a position varied among different sequencing runs and lanes (Figure S4 in Additional file 1), the ranked error rate in each subset was used rather than the absolute rate. For each position we compared the error rate of the read that mapped to the same strand in different subsets (Figure 3a, b, e, f); for both PhiX174 and mtDNA, significant positive correlations between the two subsets were observed (*P *< 0.0001; Figure 3). This is particularly true for the positions having the highest error rate, as half of the positions in the rightmost column of Figure 3a, b, e, f (which includes positions having the highest error rate in the first subset) were also included in the topmost row (which includes positions having the highest error rate in the second subset). In contrast, no specific correlations were observed between the error rate of reads mapped to different strands in the same subsets of the PhiX174 data (Figure 3c, d). For mtDNA data, a weak correlation was observed, likely to be caused by true heteroplasmic positions. When comparing our PhiX174 data with the PhiX174 data provided by Dr Ole Skovgaard, which were generated by different machines and base-calling programs, a positive correlation was observed (ρ = 0.40, *P *< 0.0001), which suggests that the sequencing error profile observed here is platform-specific (that is, Illumina-specific) or genome-specific rather than machine/chemical/base-caller-specific. Overall, these results support the interpretation that sequencing error hotspots are true features of the data, and not artifacts of particular lanes, runs, or machines.

Using the features of sequencing error described above, we developed a new method that makes use of population re-sequencing data to distinguish real mutations from sequencing errors. Since there is uncertainty regarding the error distribution among different individuals, we used three different distributions (Poisson distribution, Fisher exact test, and the empirical distribution) to calculate the bias statistic and evaluate the performance of these three methods (referred to as 'Poisson method', 'Fisher exact method', 'Empirical method'; see details in Additional file 2).

### Simulations

**Figure 4:**
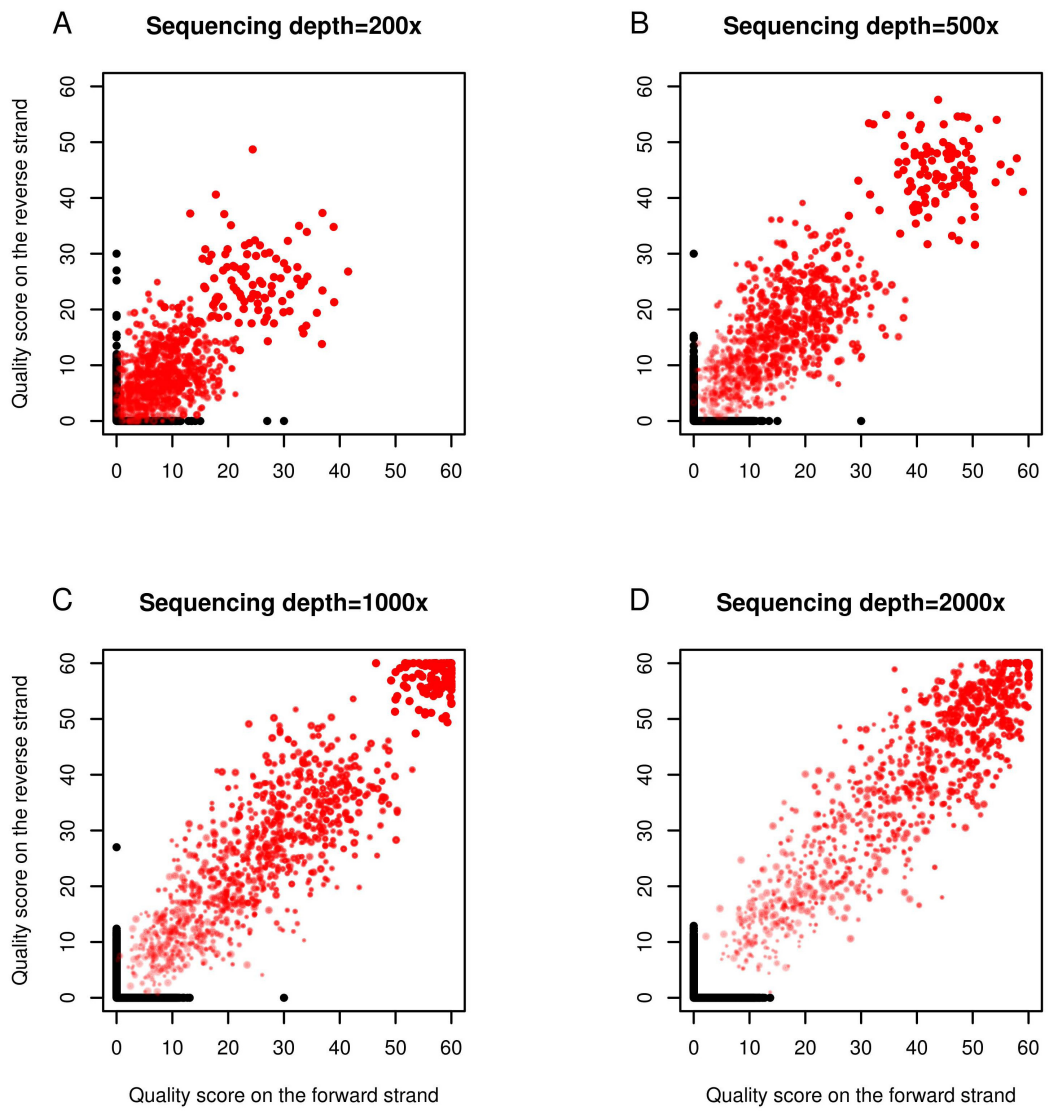
**Quality score distribution in simulations**. Quality scores are based on the Poisson method (Figures S6 and S7 in Additional file 1 for the Fisher exact and Empirical methods). Red dots represent the real LLMs, black dots represent the sequencing errors, and the size and color gradient of each dot is proportional to the frequency of the minor allele. Length of the read bins is 10 bp (see Figure S5 in Additional file 1 for results based on 5-bp read bins). **(a) **Coverage = 200×; **(b) **coverage = 500×; **(c) **coverage = 1,000×; **(d) **coverage = 2,000×.

First, we used simulations to explore the distribution of the bias statistic under different sequencing depths and mutation levels. Figure 4 shows the quality score distribution of the minor allele (both real mutations and sequencing errors) from the Poisson method. Here, quality score was positively correlated with sequencing depth and mutation level for the real mutation, but not for the sequencing error, thereby indicating that more reads and/or higher frequency of an LLM allow more accurate distinction of an LLM from sequencing error. Meanwhile, the quality score became lower for both real LLMs and sequencing errors by reducing the bin size from 10 bp to 5 bp (Figure S5 in Additional file 1), especially when the sequencing depth is low. Smaller bins exhibit larger variation (because of limited reads in each bin) and hence weaken the sensitivity of the method, and therefore are not recommended.

The Fisher exact method gave a similar quality score distribution (Figure S6 in Additional file 1) to that of the Poisson method, whereas the Empirical method showed a different distribution (Figure S7 in Additional file 1). This is because the Empirical method measures the rank of the minor allele frequency among all reference samples rather than the absolute difference between the observed minor allele frequency and that expected for an error allele. The expected quality score for an error allele was 0 for the Poisson and Fisher exact methods, but was higher and with a larger range (1 to 7) for the Empirical method. The quality score upper bound is 60 for the Poisson and Fisher exact methods, but depends on the reference sample size for the Empirical method, because the *P*-value is estimated by the rank of the observed value among all individuals (see details in Additional file 2).

Based on these results, a minimum quality score of 10 for all reads was used to distinguish LLMs from sequencing errors in our study. Applying these criteria to the simulation data results in an extremely low false discovery rate (< 1%), and an acceptable false negative rate (when sequencing depth is 500×, 50% of the rare mutations with minor allele frequency of 5% could be identified). Further details are shown in Tables S1, S2, and S3 in Additional file 3.

### Artificially mixed samples

**Figure 5:**
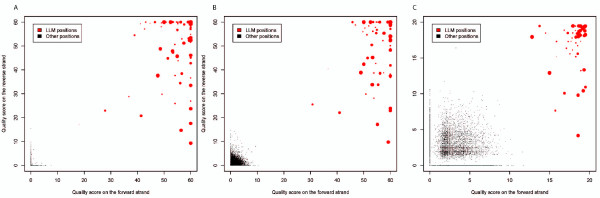
**Quality scores of the minor allele in the artificially mixed dataset**. **(a) **Quality score distribution with the Poisson method. **(b) **Quality score distribution with the Fisher exact method. **(c) **Quality score distribution with the Empirical method.

To evaluate the performance of the new method, we applied it to three artificially mixed samples, comprising a total of 78 LLMs distributed along the mtDNA genome with minor allele frequencies ranging between 3.7% to 50%. The overall coverage ranged from 138× to 4,840× (median = 1,887×), and all LLMs were successfully identified by all three methods with no false positives (Figure 5). Moreover, the minor allele quality score distributions of the three methods were similar, with the same position (position 13,708 in the 1:1 mixture, minor allele frequency of 0.42, coverage of 757×) having the lowest single strand quality score: (59,10) - with the first number the quality score for the forward strand, and the second the quality score for the reverse strand - for the Poisson method; (60,9.3) for the Fisher exact method; and (18,4) for the Empirical method. This position consistently has the lowest quality score because the minor allele is under-represented on the reverse strand: the minor allele was observed in only one bin (out of the six bins having reads) on the reverse strand, whereas it was observed in all eight bins on the forward strand.

### Comparison with other methods

We compared our method with other available methods for detecting LLMs, using the artificially mixed libraries to evaluate the performance of the other methods; the results are described below and summarized in Table 1.

**Table 1:** Comparison of the present method with other methods

Method	Parts of the read used	Thresholds	Expected haplotype number	False positives	False negatives	Reference
CRISP	All	-4; -1,000; -1,000	20	5	0	[15]
	All	-4; -1,000; -1,000	50	4	0	
SPLINTER	First 12 bp	-1.301	20	2	12	[17]
	First 70 bp	-6.6	20	123	0	
	First 12 bp	-1.301	50	2	12	
	First 70 bp	-6.6	50	634	0	
MAQ	All	60	20	848	3	[31]
	All	60	50	1,130	3	
	All	200	20	0	6	
	All	200	50	1	8	
VarScan	All	1e-10	NA	190	0	[30]
Poisson	All	10	NA	0	0	Our method
Fisher exact	All	10	NA	0	0	Our method
Empirical	All	10	NA	0	0	Our method

NA, not applicable.

CRISP, which also makes use of the population re-sequencing data, successfully identified all of the mixed positions, but also reported five false positives (when *N *= 20; four false positives when *N *= 50), which were caused by an elevated error rate in one sample (significantly different from that in other samples). Moreover, examination of the pileup file indicates that most of the error reads were located in one read bin and have the same starting coordinate on the reference genome, indicating that they probably are caused by duplicate reads.

Following the instructions for SPLINTER, we created a 2-bp context-dependent error matrix from the PhiX174 control data in the same run. When N was set to 20 and only the first 12 bp of the reads were used, 12 (18% of all the true positives) LLMs were missed, and 2 (3% of all the detected LLMs) false positives observed. A cutoff value of -2.64 removed all of the false positives but also added three extra false negatives. Increasing N to 50 did not recover the missed LLMs; examination of the pileup file revealed that these false negatives were caused by underrepresented minor alleles at the beginning of reads. We then extended the length to the first 30 bp, but 6 mutations were still missed (even with a cutoff of 0). Extending the length to 70 bp recovered all of the expected LLMs (cutoff of -6.6), but at the expense of 123 false positives.

When using VarScan, an extremely low *P*-value of 1 × 10^-10 ^identified all of the expected LLMs, but also identified 190 (71% of all the detected LLMs) false positives. When we manually refined the result by requiring double strand validation (at least three reads on each strand to call the LLM), the number of false positives was reduced to 31 (28% of all the detected LLMs). Increasing the number of reads on each strand to six left only ten (11% of all the detected LLMs) false positives.

For MAQ, a reasonable cutoff for the quality score of 60 resulted in three false negatives but thousands of false positives. Applying a stringent cutoff for the quality score of 200 removed all of the false positives but left six false negatives (7.7% of all the true positives).

In summary, none of the above methods could identify all of the LLMs in the artificial mixtures without giving false positives. To be sure, additional customized filtering or preprocessing may improve their performance. Furthermore, these methods were intended to detect non-reference alleles, rather than actually verifying the minor allele; when the reference allele is the minor allele, the *P*-value does not reflect the certainty of the minor allele, which could be problematic if the reference allele contributes to the trait investigated.

### Chimeric reads

**Figure 6:**
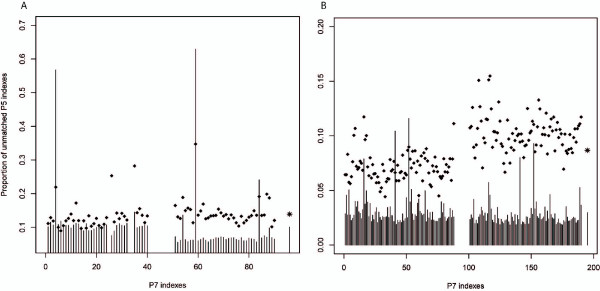
**Chimeric read fraction estimated from double indexed libraries**. P5 is the index inserted at the 5' end and P7 is the index inserted at the 3' end of the template DNA. Diamonds represent the fraction of the unmatched P5-P7 reads among all reads; the vertical line represents the fraction of the most abundant unmatched P5 component among all unmatched P5 components, while the rightmost column shows the average value of all P7 indexes. **(a, b) **The multiplexed sample sizes are 40 (a) and 90 (b), and each includes two independent libraries.

The use of double indexes (that is, indexes located at both ends of the adaptors [22]) allows chimeric reads (with mismatched indexes) to be detected. Such chimeric reads can reflect jumping PCR, index contamination, or cluster misidentification. By investigating data from four double indexed libraries, we found chimeric reads occurred at a rate of 10 to 15% when 40 to 90 samples were multiplexed and enriched for mtDNA (Figure 6). Chimeric reads could potentially result in a large number of false positive LLMs when samples with different sequences are multiplexed in one sequencing library. Here, by assuming that 15% of the reads were derived evenly from other samples in the same library, for the four libraries prepared with single indexes we found that 65 to 79% of the minor allele could be explained by chimeric reads (*P *< 10^-7^, Chi-square test), and most of them have a minor allele frequency lower than 5%. However, not all of these LLMs are necessarily caused by chimeric reads, as heteroplasmies are also prone to happen at polymorphic sites [3]. Nonetheless, these mutations were excluded from further analysis.

### Sample cross-contamination

Cross-contamination between samples is a potential problem when many samples are multiplexed in one sequencing library, or when many sequencing libraries are prepared at the same time. Although such low-level mixtures may not influence calling the consensus sequence, they may generate artificial LLMs. Our method can help to detect such cross-contamination, since the minor alleles would be identical to the major alleles in another sample.

**Figure 7:**
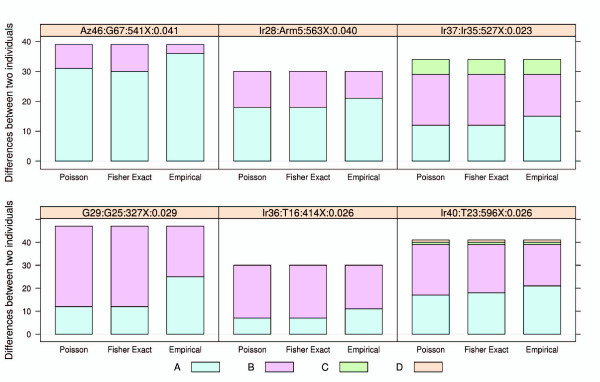
**Mixture signal in the mtDNA dataset**. The title of each plot shows the major component, minor component, average coverage, and mixture proportion. Expected minor alleles and observed minor alleles are shown in different colors. A, observed minor alleles likely due to mixture, verified by our method; B, observed minor alleles likely due to mixture, not verified by our method; C, missed minor alleles expected due to mixture; D, observed minor alleles not due to mixture.

Therefore, for all samples having more than five verified minor alleles, we examined the entire mtDNA genome dataset to see if any single sample could explain a significant fraction (≥3) of the minor alleles. Strong mixture signals were observed in two samples (Az46, Ir28) by all three methods, where all verified minor alleles (> 17) were identical to the consensus nucleotides of another sample in the same sequencing library, and represent more than 60% of the differences between the two samples (Figure 7). By averaging the minor allele frequencies at all expected variable positions, the proportions of the minor component were estimated to be 4.2 ± 0.8% for Az46 and 4.5 ± 2% for Ir28 (mean ± standard deviation).

Lower levels of contamination were detected in another four samples (Ir37, G29, Ir36, Ir40), where the mixture proportions were between 2.3% and 2.9% (Figure 7); this low mixture level makes it more difficult to recover all of the resulting LLMs. Only 37 to 53% of the expected minor alleles were identified by the Empirical method, while 23 to 43% of the expected minor alleles were identified by the Poisson and Fisher exact methods.

From the putative mixtures involving Az46 and Ir48, we could infer a false negative rate of 0.16 (11 out of 68) by the Empirical method when the minor allele frequency is around 4%. Meanwhile, from the mixtures involving Ir37, G29, Ir36, and Ir40, the false negative rate was 0.52 (79 out of 151) when the minor allele frequency is around 2.5%. The overall false discovery rate is 0.8% (1 out of 130) inferred from the 6 mixed samples, and the method could successfully detect heteroplasmy down to a level of 2.3% (Figure 7). The Poisson and Fisher exact methods both showed less sensitivity compared with the Empirical method (Figure 7). Considering the average sequencing depth of 495×, our methods showed a better performance on real data than in the simulation (Tables S1, S2, and S3 in Additional file 3). This is most likely because the empirical error rate is lower than that used in the simulations (0.0017 versus 0.01), thereby making it easier to distinguish real LLMs from sequencing errors.

In addition to these six mixtures that could be perfectly explained by contamination from one specific sample, eight samples (Arm20, Az17, Ir26, Ir29, Ir33, Ir55, Az4, Ir10) had more than five verified mutations but could only be partially explained by contamination from another sample. Therefore, they could be caused by contamination from multiple samples or by contamination from one sample as well as having true LLMs. Because of this uncertainty, we removed these samples from further analysis.

### Application to mtDNA LLM detection

By analyzing the mtDNA genome sequencing reads from 117 individuals with an average coverage of 638×, 99 LLMs were identified by the Empirical method, 63 by the Poisson method, and 60 by the Fisher exact method, with minor allele frequencies ranging from 0.5 to 49.5%. To avoid potential low-level cross-contamination from similar mtDNA genomes, we limited the analysis to mutations with a minor allele frequency of at least 5%, which then leads to the same 33 LLMs detected by all 3 methods among 30 samples (Table S4 in Additional file 4).

**Figure 8:**
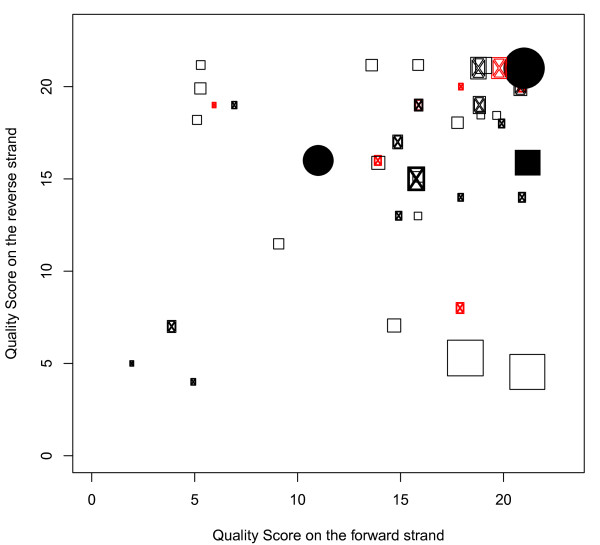
**LLMs in mtDNA detected by the new method**. Circles represent the heteroplasmies detected in our previous study [3]; squares represent the LLMs only found by our new method; circles in red color represent the LLMs that could also be explained by chimeric reads. SnaPshot validated heteroplasmies are represented by filled symbols; the size of each symbol is proportional to the minor allele frequency.

Of the 30 LLMs detected in the same individuals in our previous study, 19 were also identified by our new method (Table S4 in Additional file 4). Another seven positions had quality scores satisfying our requirement of calling LLMs but were suspected to be caused by chimeric reads (Figure 8). One position was detected in both studies but excluded here because of its lower minor allele frequency (4.6%); the frequency difference could be due to the more stringent quality control applied here or different mapping strategies (Mia versus BWA (Burrow-Wheeler Aligner)). An additional three LLMs were excluded due to low quality scores; two of these were located at position 3,492, for which all but one of the minor allele reads were mapped to the reverse strand.

All of the LLMs validated by SnaPshot assays in our previous study were recovered by our new method (Figure 8), including one position (16,223 in sample G73) that could reflect chimeric reads. If so, the mixture must have occurred during the DNA purification from the samples, as the original sample DNA was used for the SnaPshot assay. Alternatively, this may be a true LLM that happened to occur at a known polymorphic position, which in turn suggests that the LLM number reported here (33 LLMs in 117 samples) is a lower bound, as some of the LLMs excluded because they may have been caused by chimeric reads could be true LLMs.

All of the 33 LLMs involve single-base substitutions. The ratio of transitions to transversions is 10, which is not significantly different from the ratio of 8.09 for polymorphic positions in the same individuals (*P *= 0.370, Fisher exact test). The ratio of non-synonymous to synonymous LLMs in the coding region is also not significantly different from that reported in our previous study (0.5 versus 1, *P *= 0.492, Fisher exact test).

## Discussion

### Sequencing error in NGS

The development of NGS has greatly accelerated the discovery of genetic variation while significantly reducing the time and cost. However, the higher sequencing error rate of NGS also presents a computational challenge for biologists [23]. Generally, sequencing error can be categorized into two types: machine error, caused by mixed clusters, signal intensity decay, or phasing problem (for Illumina Genome Analyzer (GA)), and hence should be randomly distributed on the target sequence; and systematic error, caused by imperfect chemical/sensor/technology, which results in error rate hot spots at specific genomic locations [7,10,24].

Most of the machine error could be removed through a series of filters for quality score and/or mismatch numbers [24]. By contrast, distinguishing systematic error is much more difficult, as the quality score does not reflect the true error rate at error hotspots [7,9]. Recently, some characteristics of systematic errors have been proposed that can aid in their identification. For example, the positions G-error-G and G-error-A have the highest error rate [8], while sequencing error hot spots tend to be located after inverted repeats and GGC or GGT sequences [7,9,10]. Although we also found that these features are correlated with systematic errors statistically, they can only explain a limited portion of the error rate variance, as the position following these motifs showed an error rate with up to ten-fold variation (Figure S3 in Additional file 1), as also noted recently elsewhere [10].

In our study, we found that sequencing errors often occur at the same position in different individuals, especially for positions with the highest error rate (Figure 3). This tendency is unlikely to be caused by the particular sequencing machine because the correlation could also be observed between the data generated by difference machines. This observation suggests that the error rate for a sequence of interest could be estimated from a reference panel having the same sequence. Moreover, by examining the error rate for different parts of the reads and for reads from different strands, after our quality filter we found that: 1) sequencing error varies across different parts of the read and at different positions (Figure 2; Tables S5 and S6 in Additional file 3); and 2) the error rate is strand-specific (Figure 2), as sequencing contexts on the two strands are different most of the time. Therefore, the position and orientation of the reads mapped to the questioned position should be considered when estimating the sequencing error rate.

Another issue is whether or not to remove duplicate reads, as these may reflect the same starting template molecule. On the one hand, including duplicate reads could amplify the error signal arising from PCR errors, but on the other hand, removing duplicate reads based only on the starting coordinate on the genome leads to a significant reduction of coverage (for single-end reads). By analyzing the paired-end data from two mtDNA sequencing libraries with equivalent sequencing depths, we found each segment in the library was duplicated an average of 1.19 times, with 454 segments (0.01%) duplicated more than 10 times, and the most duplicated segment present in 247 copies. For the artificially mixed samples, by removing the duplicate reads, we lost more than 90% of the reads and 4 LLM positions were missed by our method. Therefore, it would be reasonable to keep all the reads in the analysis while taking duplicate reads into consideration when identifying the LLM.

### Features of our method

Understanding the sequencing error makes it possible to distinguish errors from real LLMs. While various methods are available that utilize different features of sequencing errors, we have developed a method that performs better than other methods in detecting LLMs (based on artificially mixed samples, where the sequencing depth/minor allele count is much lower than that used/suggested in other studies [14,17]). Moreover, the available standard genotyping methods are not designed for LLM detection. For example, for the widely used GATK toolkit [11] there are only three possible allele frequencies (0%, 50%, 100%), whereas there is a much wider range of frequencies for LLMs. Moreover, GATK requires a reliable SNP database, which does not exist for LLMs, and GATK makes use of metrics to assess/refine the new SNP calls (such as the ratio of transitions to transversions) that do not exist for LLMs.

The method introduced here is based on several features. First, our method estimates the error rate from population re-sequencing data. For each position in the target region, the empirical error rate is estimated from all reads mapped to the reference samples that have the same consensus nucleotide. Therefore, we use the complete sequencing context, rather than a more limited or entirely different context, to estimate the sequence error. Moreover, since all samples are analyzed following the same pipeline, any errors introduced during the analysis (such as mapping error) are also taken into consideration.

Second, the distribution of the reads is taken into account. As shown above, not all reads mapped to the same position have the same error rate; thus, we categorize the reads into bins according to their position and orientation mapped to the target sequence. As the error rate in each bin is estimated separately, reads in different bins give different weights in calling LLMs. In addition, the contribution of each bin to the final quality score has an upper limit, to prevent false positives caused by duplicate reads.

Third, an absolute frequency or coverage cutoff is not required. A frequency threshold is widely used to distinguish LLMs from sequencing errors, but such a frequency threshold should be coverage-dependent, that is, the frequency threshold becomes smaller with higher coverage. Since the sequencing depth is unevenly distributed along the genome, a single frequency threshold would either overestimate or underestimate the true number of LLMs. Instead, in our method a *P*-value is calculated to represent the likelihood of the observation under the null hypothesis (minor allele is caused by sequencing error).

Fourth, our method gives an understandable Phred-like quality score, which reflects the reliability of the minor allele for each position. This makes it easier to apply different discovery strategies, depending on the wishes of the investigator, that is, a higher false positive rate with a lower false negative rate, or a lower false positive rate with a higher false negative rate.

### Flexibility of our method

Ideally, the reference samples used to estimate the error rate should not have any LLMs, or only a small number of LLMs at the same position. In practice, this assumption holds at most LLM positions; however, for common variation where a significantly higher error rate would be observed in most bins, a fixed error rate could be used (as implemented in the Poisson method). For example, by using an error rate of 0.01 when the reference error rate is significantly higher than 0.01, we successfully detected all of the common variations in the PhiX174 dataset without any false positives (Figure S8 in Additional file 1). However, if reference samples are lacking for the region of interest, an error rate estimated from control data, quality score, or some other dataset for all the positions and all bins could be used. In the present study, we did not observe any error hotspot having an error rate significantly higher than the overall error rate in our method (that is, that passed our threshold to call a LLM). However, using an averaged error rate may result in a higher false negative rate.

Due to the uncertainty of the underlying distribution of sequencing error across the target sequence, we introduced three methods to calculate the *P*-value of the deviation of the observation from expectation. The Poisson method assumes the sequencing error rate follows a Poisson or binomial distribution, whereas the Fisher exact and Empirical methods do not assume any specific distribution for sequencing errors. The Poisson and Fisher exact methods measure the absolute difference between the observed minor allele frequency and the error rate, whereas the Empirical method measures the ranking of the minor allele frequency among all reference error rates. In our study, all three methods showed good specificity (false discovery rate < 1%). The Empirical method has a higher sensitivity when the minor allele frequency is low (< 5%), in which case the difference between the minor allele frequency and error rates tends to be amplified by ranking the minor allele frequency (for example, the minor allele frequency that ranked first could still be very close to the remaining observations). However, the Empirical method should be used with caution when processing the data from different sequencing lanes/runs, as an intrinsic sequencing error difference could exist between reference samples and test samples due to the variation among lanes/runs (Figure S4 in Additional file 1), and such a spurious difference may be captured as a signal of LLM.

Although the data for this study came solely from the Illumina platform, the input to our pipeline is a SAM file [25], thus making it feasible to process the data from any platform for which the data can be converted to SAM format. It is also straightforward to implement other customized processes (for example, base quality score recalibration or re-alignment) before applying our method. Although the sequencing error profile varies substantially among different technologies/base-callers, our method does not require any prior knowledge of the error profile, as all relevant information is extracted from the entire re-sequencing dataset.

A further application of the method could include standard genotyping estimation for diploid sequences. However, several issues would need to be addressed, including: how to estimate the error rate when heterozygotes are considered; how to bin the reads when the coverage is low; and how to calculate the quality score for heterozygotes.

#### Other problems in detecting LLMs

Sequencing error is not the only issue in detecting LLMs. Cross-contamination is another major issue, especially when handling large numbers of samples simultaneously. Although normally the contamination fraction is very small, the nucleotide derived from the minor contamination component behaves exactly the same as an LLM. Hence, it is impossible to distinguish a contamination allele from a true LLM. Here, we provide a straightforward way to identify contamination: after producing the list of potential LLMs we can infer the contamination based either on the total number of minor alleles (if these exceed some expected value), or similarity to other samples in the same library, or in other libraries, or in databases. For example, with the mtDNA genome sequencing data, if more than five LLMs are detected in a sample, it would be suspected to be a mixture, because it is unlikely for a single individual to harbor more than five heteroplasmic positions [1,3,12]. For such suspected mixtures, we then examine other sequences from the same library (as well as from other libraries prepared at the same time) to determine if the LLM component could be explained by mixture from a specific sample. We also use databases such as Phylotree [26] to determine if the minor alleles are likely to come from one specific haplogroup. In our study, we could detect contamination down to 2 to 3%, and almost half of the expected minor alleles are accurately recovered at that level with an average coverage of approximately 500×. This suggests it would be possible to find contamination at a lower level with higher coverage. However, the ability to detect contamination relies on the number of variable positions between the samples that contribute to the mixture: if they are very similar, then it would be very difficult to tell whether it is real LLM or contamination. Examination of other genomic regions would be needed.

Chimeric reads are also a potential problem with multiplex sequencing, as then reads not only come from the target sample, but also from other samples in the same sequencing library. Double indexes allow chimeric reads to be detected, and by applying double indexes in four libraries, we found 10 to 15% of the reads to have mismatched indexes (Figure 6). This is much higher than the 0.3% reported previously [22], possibly because the cluster density in our study was 1.5-fold higher than in theirs and more (heterogeneous) samples were multiplexed in our libraries. Index contamination is another potential source of chimeric reads, but unlikely to be a contributing factor in our study because the unmatched P5 indexes seem to be randomly derived from other indexes (Figure 6). By considering the library composition at each position, we found up to approximately 70% of the minor allele could be explained by chimeric reads. Although not all of the LLMs are false positives, chimeric reads remain a serious concern, and double indexes are advised.

## Conclusions

Due to the higher sequencing capacity and reduced cost, NGS is now used widely, especially in discovering genetic variation in populations. There is great interest in using NGS to detect LLMs, especially in such applications as mtDNA heteroplasmy, characterizing tumors, and pooled samples. However, distinguishing sequencing error from LLM remains a major challenge. Here, we provide a novel computational method to distinguish LLM from sequencing error and apply this to mtDNA genome sequence data. Although the method is highly efficient at detecting LLMs, not all of these are necessarily real LLMs, because they may also be caused by contamination or chimeric reads. To exclude these other possibilities, not only efficient algorithms but also more refined experiment protocols are needed.

## Materials and methods

### Data

A PhiX174 bacteriophage shotgun library with a specific index is routinely spiked in and sequenced in each lane of our in-house runs of the Illumina GAIIx and then used as the training dataset for the IBIS base-caller [27]. Here, we retrieved 17 million PhiX174 reads from 35 lanes (76-bp single-end reads), with an average sequencing depth of 193,874×. Another PhiX174 dataset (34-bp single-end reads, 32,473×) was kindly shared by Dr Ole Skovgaard, and was used to compare the sequencing error profile between different sequencing machines based on the same platform. A third PhiX174 dataset of 26 million reads was obtained from a control lane accompanying the 76-bp mtDNA data; this dataset was used to estimate the error matrix needed by SPLINTER. Although the PhiX174 data are thought to come from a single strain, we and others have previously observed two LLMs located at positions 1,401 and 1,644, both with minor allele frequencies of around 30% [3].

The mtDNA genome sequence data from our previous study [3], which includes 131 individuals, was obtained by sequencing long-range PCR products in 4 Illumina GAIIx lanes following a multiplex protocol. The average sequencing depth varied from 457× (36-bp read) to 1,879× (76-bp read) per individual. Sequencing data from another two mtDNA paired end libraries were used to estimate the fraction of duplicate reads in our study, and data from four mtDNA libraries prepared with double indexes were used to estimate the proportion of reads coming from two different templates.

The data for artificially mixed samples also came from our previous study [3], where we mixed template DNAs from two individuals differing at 25 positions (the individual whose DNA was used as the major component has a heteroplasmy at position 13,604, with a minor allele frequency of around 15%). The two DNA samples were mixed at ratios of 1:1, 1:3, and 1:9.

Raw sequencing data are publicly available from the European Nucleotide Archive's Sequence Read Archive through accession numbers ERP000879 (mtDNA reads) and ERP001254 (PhiX174 reads).

### Quality control

IBIS was used to recalibrate the quality score generated by the Illumina base-caller, Bustard [27]. Raw reads having more than 5 bases (2 bases for 36-bp reads) with quality scores of 15 or less were removed from the analysis, and the adaptor sequences were trimmed. PhiX174 and mtDNA reads were then mapped to [GenBank: NC_001422] and [GenBank: NC_012920], respectively, using BWA (Burrow-Wheeler Aligner) [28]. Reads with mapping scores < 20 or mismatch numbers > 2 were removed and output files were further converted to pileup format using Samtools [25]. For calling LLMs, only bases with quality scores of 20 or more were used.

### Algorithm to call LLMs

We refer to the nucleotide position to be investigated for LLMs as the target position. For each individual, reads that included the target position were assigned into different read bins according to the strand they mapped to and the mapped position within the reads. For the latter, reads were divided into 10-bp segments; thus, with a dataset of 76-bp reads, there are 16 read bins (8 segments × 2 strands). To call an LLM at the target position in any individual, all other individuals with reads that include the target position are used as the reference. The sequencing error profile in each read bin for the target position was estimated from reference individuals with the same consensus nucleotide at the target position (this step was omitted if the same nucleotide was observed in 50 or fewer reference individuals). A *P*-value was calculated by comparing the observed and the reference error profile in each read bin (null hypothesis: there is no difference between the questioned individual and reference individuals). This *P*-value was used to calculate the bias statistic, which measures the deviation of the observed minor allele count from the expected error count. Since the underlying distribution of the sequencing error among different individuals is unknown, the *P*-value was estimated in three different ways and their performance was evaluated: (1) Poisson *P*-value; (2) Fisher exact *P*-value; (3) Empirical *P*-value. Details as to how these are calculated are described in Additional file 2.

The bias statistic can be easily converted to a Phred-scaled quality score, which reflects the uncertainty in calling the LLM: a higher value demonstrates support from more read bins and a larger deviation from the reference panel, while lower values reflect smaller deviations from the reference panel.

Scripts can be freely obtained from the DREEP website [29].

### Simulation framework

To evaluate the sensitivity and specificity of our method for the detection of LLMs, we performed simulations representing different mutation levels and different sequencing depths. Additionally, to investigate the effect of the length of the read bin, two lengths were used: 5 bp and 10 bp. For each simulation, the target region was 16,569 bp, the sequencing error rate was set to 1% and the length of the reads was 76 bp. The LLM level (minor allele frequency) was set to 2%, 3%, 4%, 5%, 6%, 7%, 8%, 9%, 10%, or 20%, the position of the LLM was assigned randomly on the sequence by generating a random number among all possible positions. Reads were generated using Wgsim (0.1.18) [25]. For each condition, the number of reads was varied (40 k, 100 k, 200 k, 400 k) in order to simulate different sequencing depths (200×, 500×, 1,000×, 2,000×), and for each setting, the simulation was repeated 100 times.

### Sequencing error hot spots and cold spots

We used the following criteria to define sequencing error hot spots in the PhiX174 sequences: the sequencing depth was at least 10,000× and the probability of observing a count equal to or greater than the observation should be less than 1/Length of target genome/1,000,000 under a Poisson distribution (with mean equal to the average error rate estimated from all positions and reads). Also, error hot spots are defined to be strand-specific (to avoid possible common variation positions), with the error rate at least ten times higher on one strand than on the other strand. We also defined sequencing error 'cold spots' as positions where the error rate on one strand was less than half of that on the other strand and the probability of observing a count equal to or less than the observation should be less than 1/Length of target genome/1,000,000 under a Poisson distribution.

### Comparison with other methods

In order to compare the performance of the method described here with other available methods, the following software were applied to detect LLMs in the artificial mixture dataset: CRISP (v5) [15], SPLINTER (6o) [17], VarScan (2.2.5) [30], and MAQ (0.7.1) [31]. For software that requires the expected haplotype number (CRISP, SPLINTER, MAQ), 20 and 50 haplotypes were both used. Different *P*-values or quality score cutoffs were tried, and the one giving the best result (that is, lowest false positive and false negative rates) was chosen. For CRISP, the first *P*-value was set to be -4.0, the other two *P*-values were less than -1,000, the minimum quality score was 20, the minimum mapping score was 20, the maximum mismatch number was 2; all of these were identical to the parameters used in our method. For SPLINTER, the *P*-value cutoff was set to be 0.05 (-1.301) when the first 12 bp was used and the error matrix was estimated from the PhiX174 data generated in the same run. For VarScan, the minimum number of reads to call a SNP was 30 (1.5% of the sequencing depth), the minimum variant frequency was 2%, the minimum quality score was 20, and the *P*-value cutoff was set to be 1 × 10^-10^. For MAQ, the quality threshold for the final SNP was set to be 60 and 200, and -E = 0 was set to call a LLM, and -D was changed to 5,000 in order to avoid any false negatives due to the high sequencing depth. Since all of these programs are designed to detect the non-reference allele, we changed the reference sequence to the consensus sequence of the individual who contributed the most reads to the mixed pool; thus, all of the LLMs detected should be derived from the minor component.

## Abbreviations

bp: base pair; GA: Genome Analyzer; LLM: low level mutation; mtDNA: mitochondrial DNA; NGS: next-generation sequencing; PCR: polymerase chain reaction; SNP: single-nucleotide polymorphism.

## Competing interests

The authors declare that they have no competing interests.

## Authors' contributions

Programming and analyses were performed by MK with input by MS. The manuscript was written by MK and MS. All authors read and approved the final manuscript.

## Supplementary Material

Additional file 1 **Supplemental figures**. This file contains Figures S1, S2, S3, S4, S5, S6, S7, and S8. Click here for file

Additional file 2 **Supplemental materials and methods**. Supplemental materials and methods include the following sections: Calculation of bias assuming a Poisson distribution; Calculation of bias using the Fisher exact test; Calculation of bias using the empirical distribution; Refinement of the method. Click here for file

Additional file 3 **Supplemental tables**. This file contains Tables S1, S2, S3, S5, and S6. Click here for file

Additional file 4 **Table S4 - LLMs detected in the mtDNA dataset**. An EXCEL table listing all the LLMs detected in the mtDNA dataset. Click here for file
