# Supplementary material for: A new approach for detecting low-level mutations in next-generation sequence data
Source: Genome Biol. 2012 May 23;13(5):R34. doi: 10.1186/gb-2012-13-5-r34 (PMC3446287; doi:10.1186/gb-2012-13-5-r34)

Additional data file 1

Supplemental Figure 1. Sequencing error rate along the genome

Sequencing error rates are represented as Phred quality scores.

Supplemental Figure 2. Sequence motifs preceding the sequencing error hot spots and cold spots on the PhiX174 genome

WebLogo was used to identify the 10-bp sequence motif preceding the sequencing error hot spots (A) and cold spots (C). Motifs in different orientations are showed separately (F, forward; R, reverse). The sequencing error rate for positions following the same motif are shown in B, D. Black color dot refers to the motif located on the forward strand; blue color dot refers to the motif located on the reverse strand in B, D.

Supplemental Figure 3. Sequencing error rate following 2-bp motifs

Only the first part of each read ($2^{nd} \sim 11^{th}$ positions) was used to estimate the sequencing error rate.

Supplemental Figure 4. Sequencing error rate for different parts of the read and in different lanes and runs.

The X-axis indicates the strand and read bin.

Supplemental Figure 5. Quality score distribution in simulations, Poisson method (5bp bin).

Red dots represent the true LLM, black dots represent the sequencing errors, and the size and color gradient of each dot is proportional to the frequency of the minor allele.

Supplemental Figure 6. Quality score distribution in simulations, Fisher Exact method (10-bp bin).

Red dots represent the true LLM, black dots represent the sequencing errors, and the size and color gradient of each dot is proportional to the frequency of the minor allele.

Supplemental Figure 7. Quality score distribution in simulations, Empirical method (10-bp bin).

Red dots represent the true LLM, black dots represent the sequencing errors, and the size and color gradient of each dot is proportional to the frequency of the minor allele.

Supplemental Figure 8. Quality score for common variation in the PhiX174 dataset

Red circles represent the common variants and black circles represent the sequencing errors; the size of each circle is proportional to the frequency of the minor allele. Length of the bins is 10-bp.

Supplemental fig 1

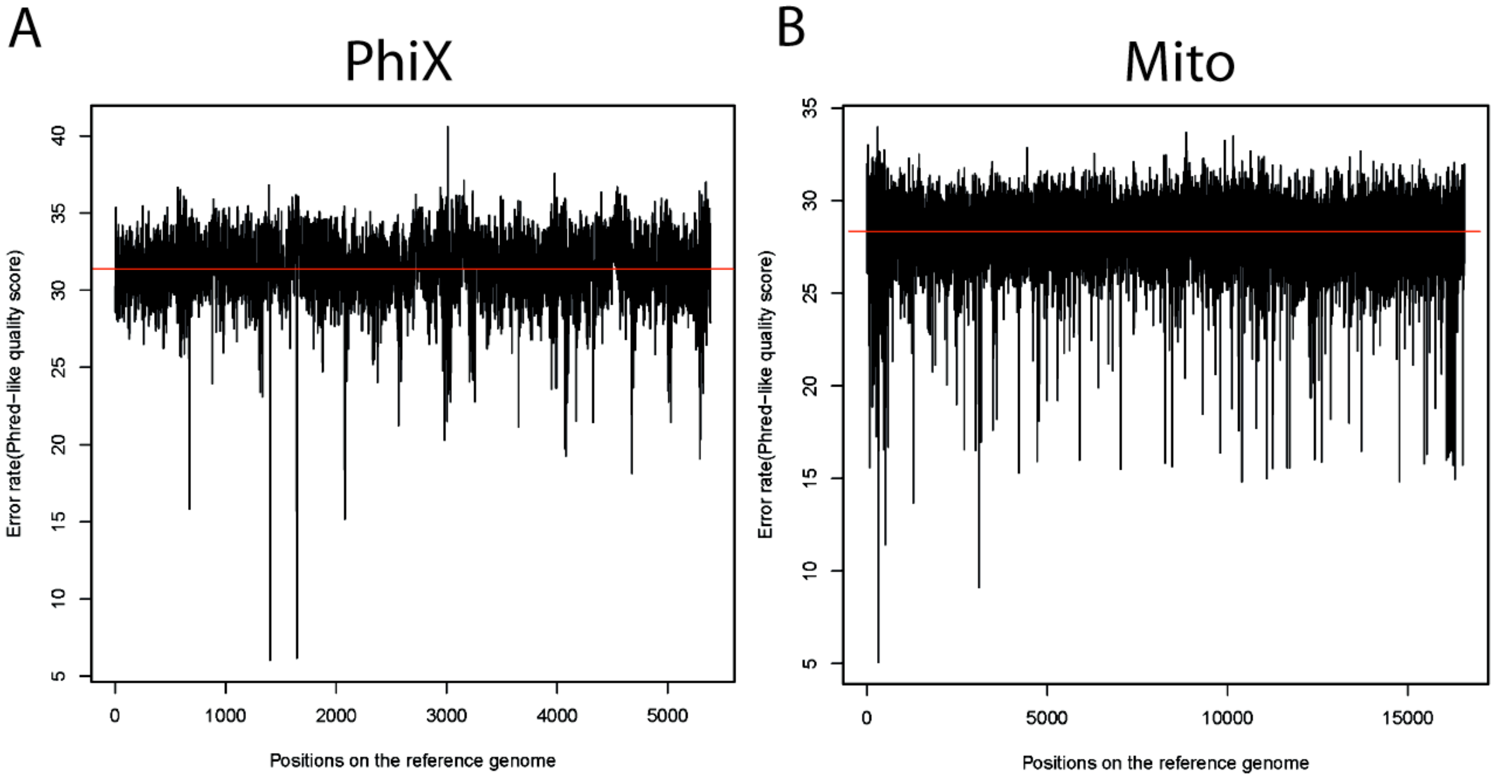

Supplemental fig 2

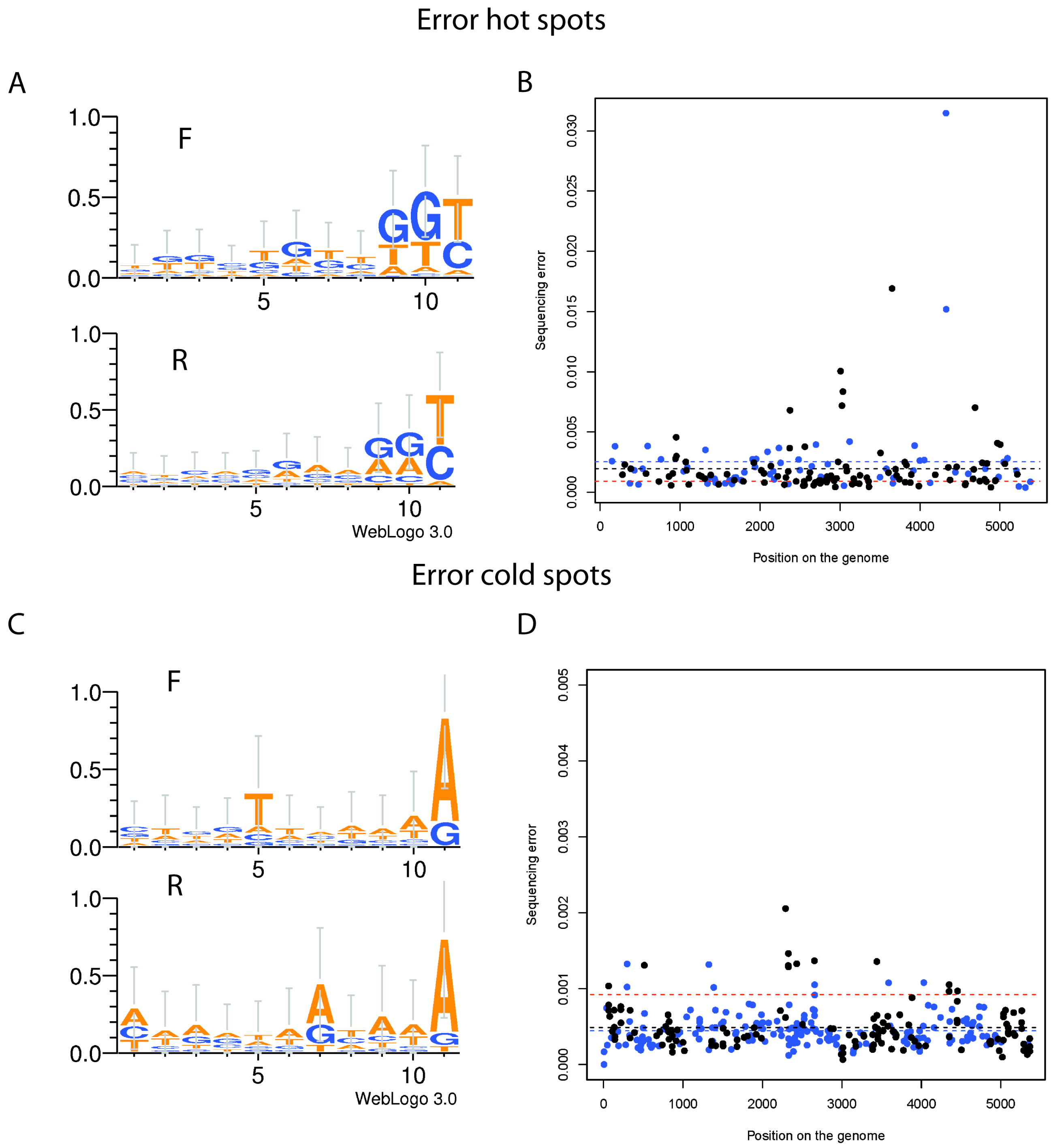

Supplemental fig 3

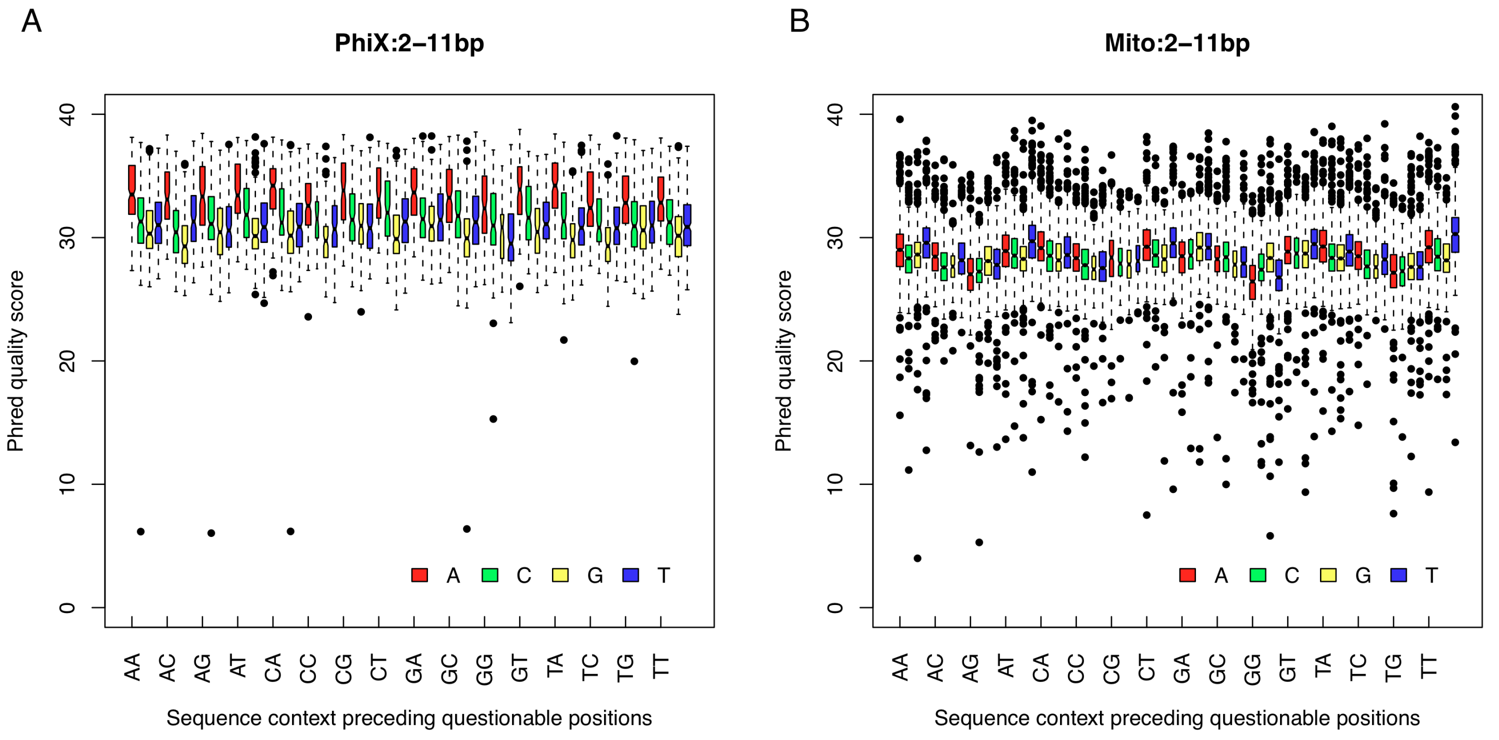

Supplemental fig 4

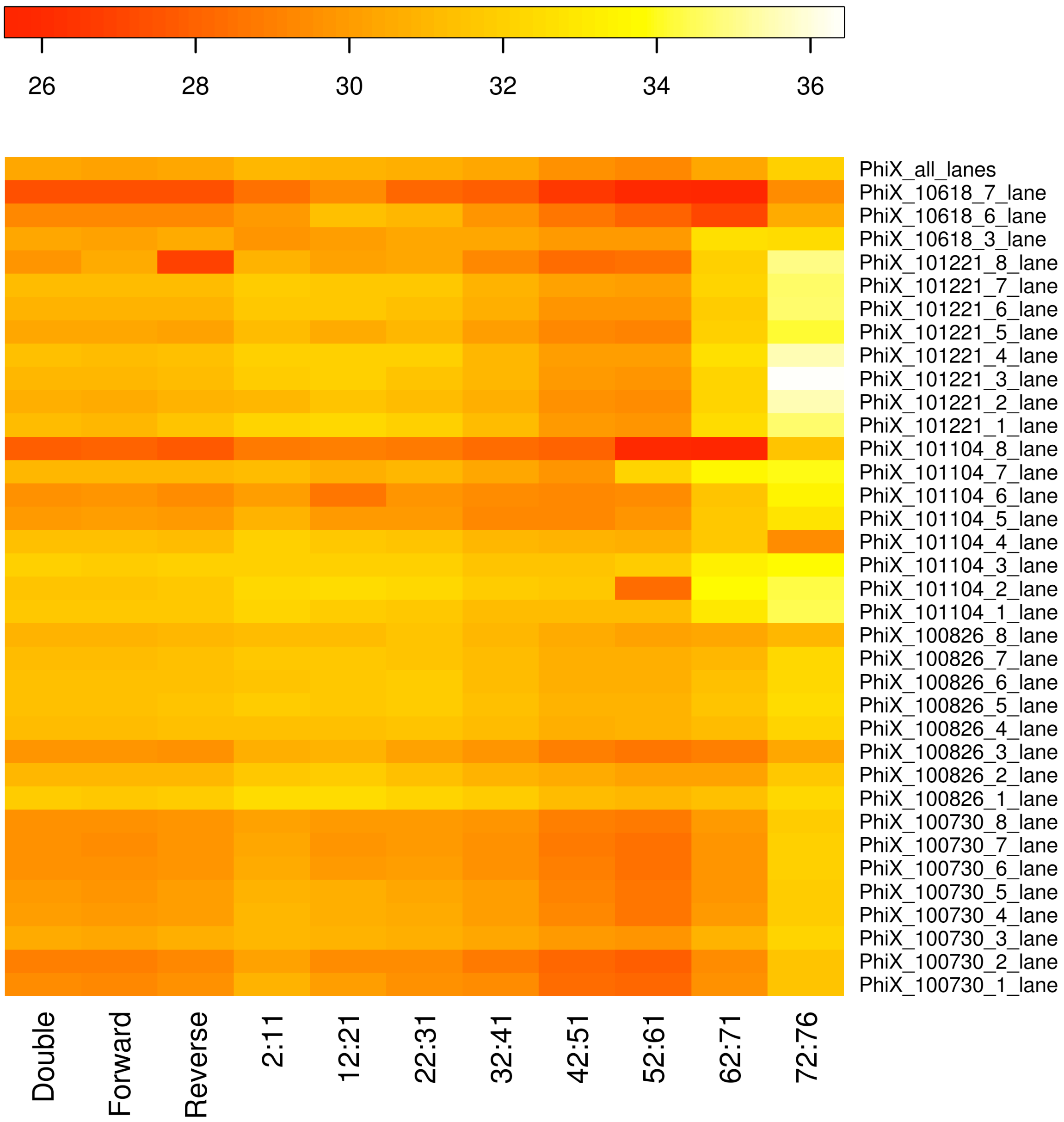

Supplemental fig 5

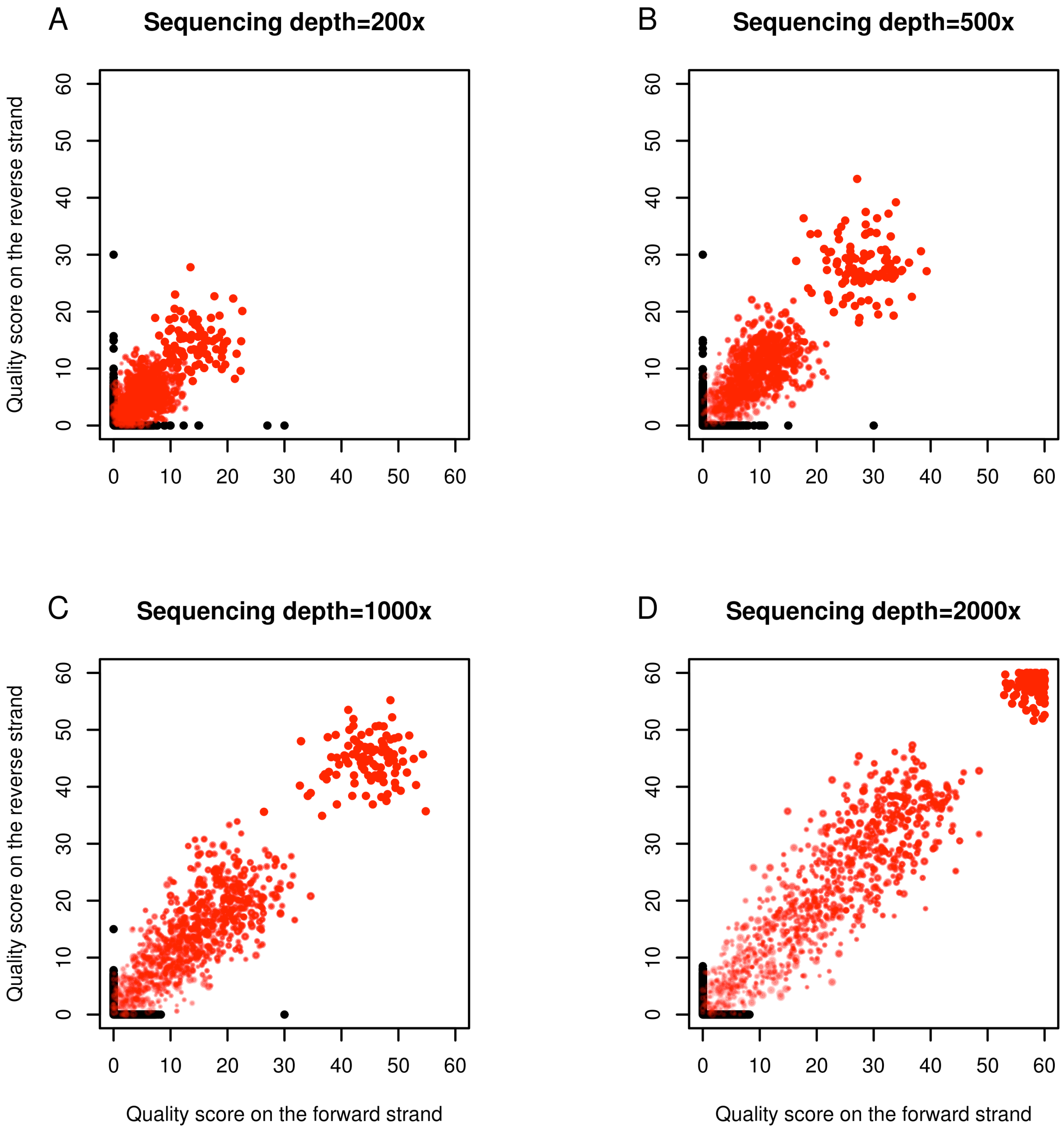

Supplemental fig 6

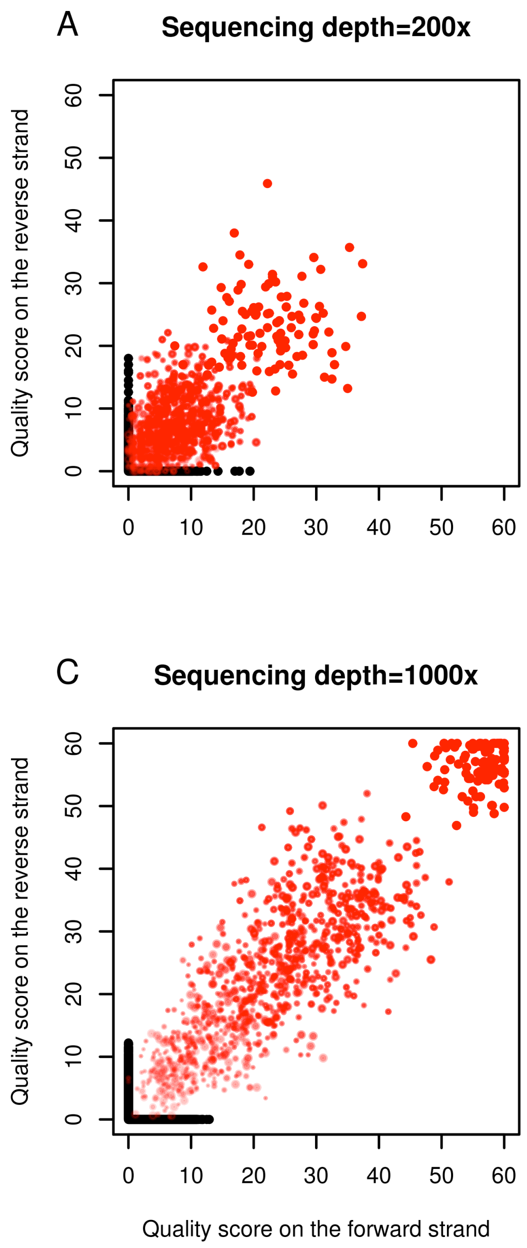

Supplemental fig 7

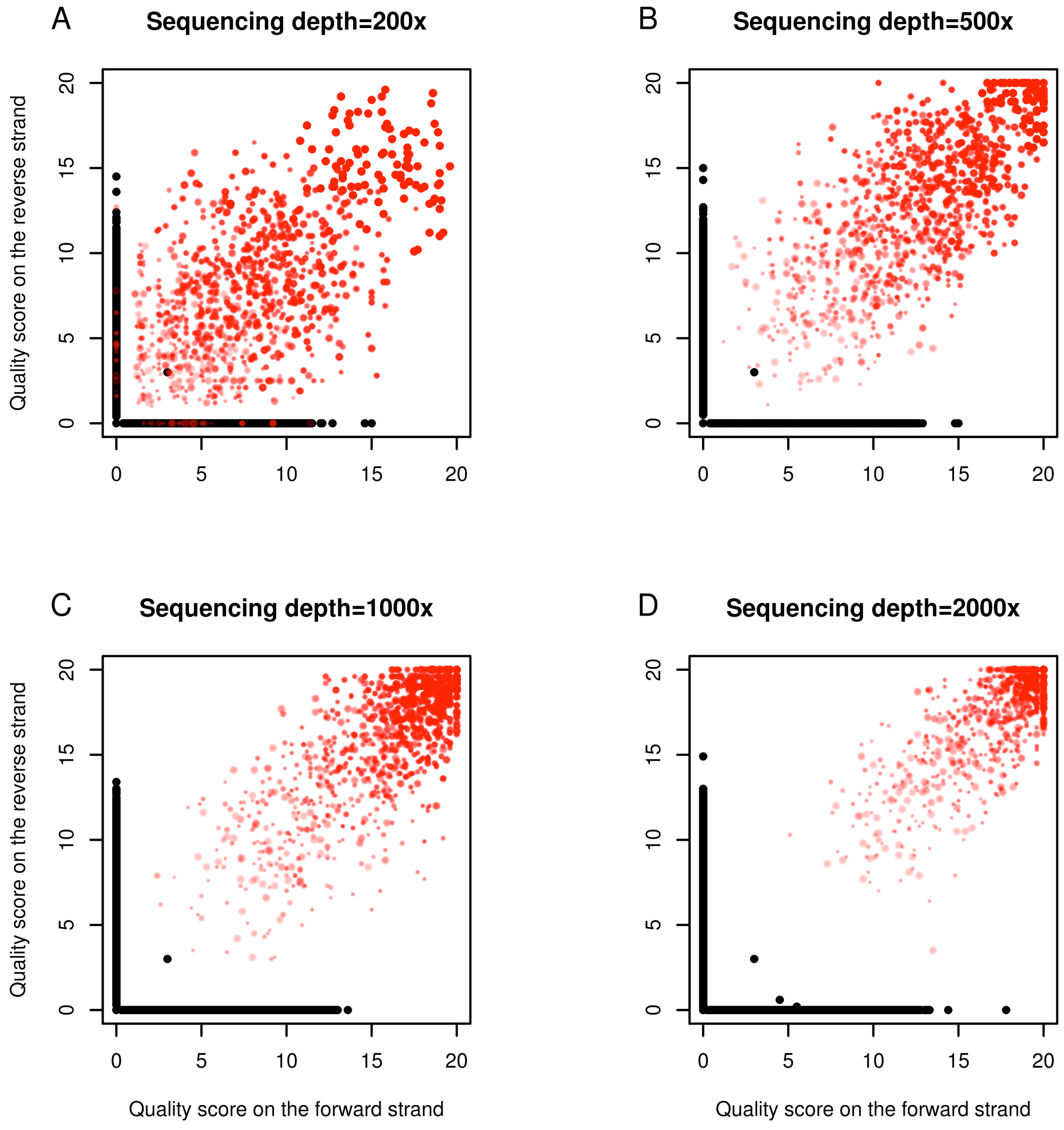

Supplemental fig 8

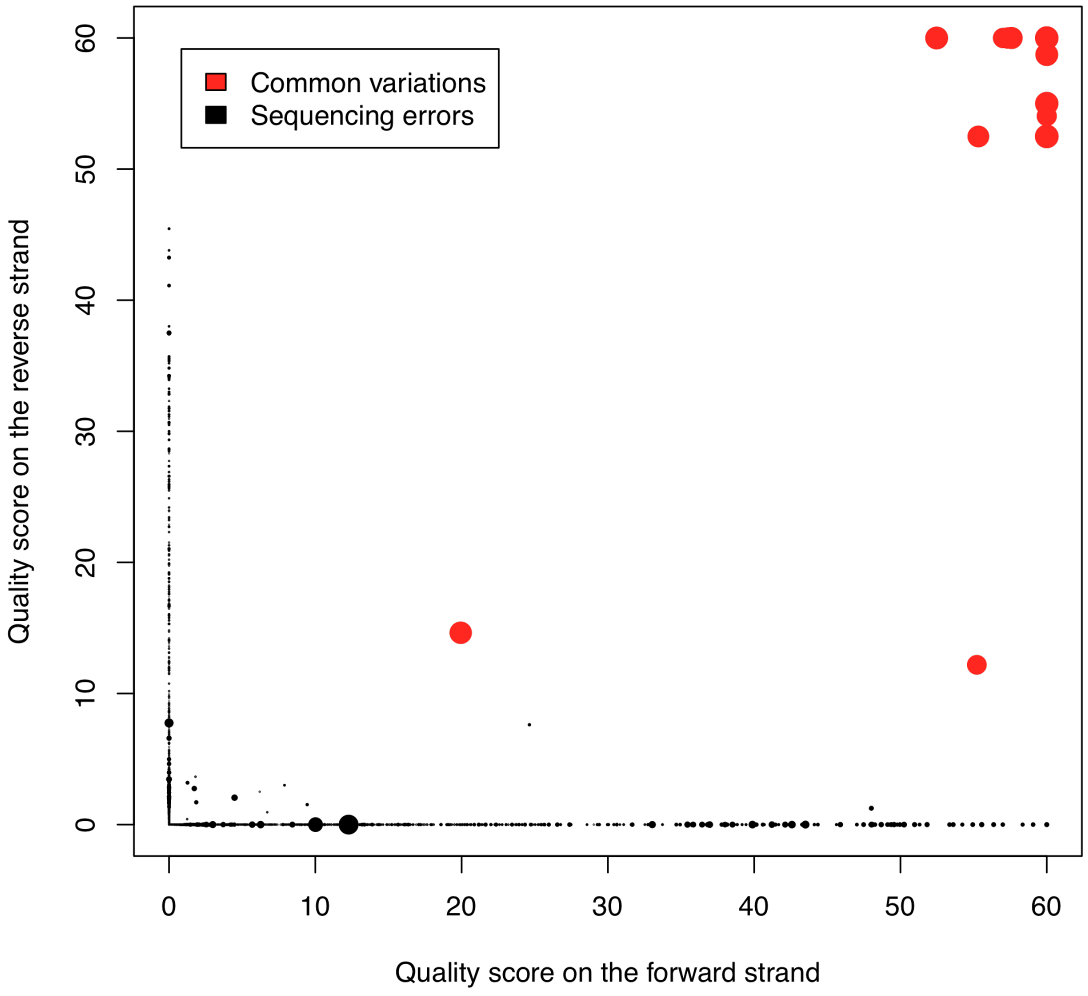

Supplement: Additional file 1 — Supplemental figures. This file contains Figures S1, S2, S3, S4, S5, S6, S7, and S8. [file gb-2012-13-5-r34-S1.PDF]
