# Supplementary material for: A new approach for detecting low-level mutations in next-generation sequence data
Source: Genome Biol. 2012 May 23;13(5):R34. doi: 10.1186/gb-2012-13-5-r34 (PMC3446287; doi:10.1186/gb-2012-13-5-r34)
Supplement: Additional file 2 — Supplemental materials and methods. Supplemental materials and methods include the following sections: Calculation of bias assuming a Poisson distribution; Calculation of bias using the Fisher exact test; Calculation of bias using the empirical distribution; Refinement of the method. [file gb-2012-13-5-r34-S2.DOC]

**Additional data file 2**

**Supplemental materials and methods**

**Calculation of Bias assuming a Poisson distribution**

The sequencing error rate for each position was estimated for each read bin that overlapped the position from reference individuals having the same consensus nucleotide at that position. For the *i-th* position, with consensus nucleotide *c*, the error rate on strand *d* and in the *s-th* segment of the read was calculated as:

$$error\left(i,c,d,s\right)=\frac{\sum_{ref\text{ }indi(consens=c)} n(i,!c,d,s)}{\sum_{ref\text{ }indi(consens=c)} n(i,!c,d,s)+n(i,c,d,s)}$$

*n(i,c,d,s)* is the number of reads which were mapped to strand *d* with segment *s*  mapped to position *i* on the reference sequence, and giving a *c* allele at position *i*. By contrast, *n(i,!c,d,s)* represents the number of reads without the c allele at position *i* on the reference sequence.

When detecting LLM, for each read bin at a specific position, the expected error count was calculated by multiplying the sequencing depth in the bin by the empirical error rate for the bin. *Bias* was then estimated by combining the information from all bins:

$$Bias=\frac{\prod_{read\text{ }bin(Obs>Exp)} P\left(N\geq Obs|Obs>Exp\right)}{\prod_{read\text{ }bin(Obs\leq Exp)} P\left(N\leq Obs|Obs\leq Exp\right)}$$

$P\left(N\geq Obs|Obs>Exp\right)$ denotes the probability of observing an occurrence equal to or greater than the observation under the Poisson distribution when the observation is greater than expectation. Similarly, $P\left(N\leq Obs|Obs\leq Exp\right)$ denotes the probability of observing an occurrence equal to or less than the observation under the Poisson distribution when the observation is equal to or less than the expectation.

Under the null hypothesis that the observed minor allele could be explained by sequencing error under the Poisson distribution with λ ≤ empirical error rate (which is used as the mean value when calculating $P\left(N\geq Obs|Obs>Exp\right)$ and $P\left(N\leq Obs|Obs\leq Exp\right)$ ), where λ is the mean value for the real error rate (which could be equal to or less than the empirical error rate, due to the possible LLMs in the reference panel), we infer that:

$$\begin{matrix} \left\{\begin{matrix} P(Obs>Exp)\approx P(Obs\leq Exp) \\ P\left(N\geq Obs|Obs>Exp\right)\approx P\left(N\leq Obs|Obs\leq Exp\right) \end{matrix}\right.\text{ }if\text{ }\lambda=empirical\text{ }error\text{ }rate \\ \left\{\begin{matrix} P(Obs>Exp)<P(Obs\leq Exp) \\ P\left(N\geq Obs|Obs>Exp\right)>P\left(N\leq Obs|Obs\leq Exp\right) \end{matrix}\right.\text{ }if\text{ }\lambda<empirical\text{ }error\text{ }rate \end{matrix}$$

Thus, *Bias* would be equal to or greater than 1. On the other hand, if λ > expected error count, thereby indicating that the minor allele could not be explained by the empirical error rate, we would have:

$$\left\{\begin{matrix} P(Obs>Exp)>P(Obs\leq Exp) \\ P\left(N\geq Obs|Obs>Exp\right)<P\left(N\leq Obs|Obs\leq Exp\right) \end{matrix}\right.\text{ }if\text{ }\lambda>Exp$$

Under this case, *Bias* would be less than 1.

In order to make the *Bias* more understandable, we convert it to a Phred-like quality score:

*n* is the number of read bins used in the previous calculation, which is equal to or less than the number of theoretical read bins (because some bins may not have any reads). Quality scores, which reflect the deviation of the observed minor allele from the expectation under the error model, are calculated for double strands and for each single strand for the convenience of further filtering. The quality score is positive when the observed minor allele count is greater than the expected error count, otherwise, it is 0 or a negative value (which was then converted to 0).

**Calculation of Bias using the Fisher Exact test**

**For each bin at a specific position, we count the number of reads with the major allele *a* and the minor allele *b* for the individual in question, as well as the same numbers *c, d* for the reference individuals, and then create a 2×2 contingency table:**

|  | Questionable individual | Reference individuals |
| --- | --- | --- |
| Major allele | *a* | *c* |
| Minor allele | *b* | *d* |

The p-value was calculated by two Perl modules Text::NSP::measures::2D::Fisher2::left and Text::NSP::measures::2D::Fisher2::right (<http://search.cpan.org/dist/Text-NSP/>), and minor allele frequencies were calculated for both the individual in question (*fq*) and reference individuals (*fr*) in each bin. *Bias* was calculated as:

Bias was then converted to a Phred-like quality score as above.

**Calculation of Bias using the empirical distribution**

For each bin at a specific position, we first calculated the minor allele frequency for the individual in question (*fq*) and compared this to the minor allele frequencies of all the reference individuals with the same consensus nucleotide. We then obtained the number of reference individuals having a minor allele frequency *fr* equal to or greater than *fq* (*N(frfq)*=1 if *fq* is the largest) . The p-value was then calculated as:

$$p-value=\frac{N(fr\geq fq)}{N}$$

where *N* is the total number of reference individuals. Then,

$$Bias=\prod_{read\text{ }bins} p-value$$

Bias was converted to a Phred-like quality score as described above. Note that *Bias* here is the product of p-values of all read bins, rather than a ratio as in the previous methods; therefore, the expected Phred-like quality score under the null hypothesis would be 1~7 rather than 0.

**Refinement of the method**

All reads were used in this analysis, thus, duplicate reads could be problematic at some positions. To solve this problem, a lower bound of 1e-6 was given for the p-value to reduce the weight of any single read bin. This also results in a maximum quality score of 60 for the first two methods (the upper bound of the Empirical method depends on the reference sample size).

For the individual with a rare consensus base in the reference dataset, the error profile could be seriously biased by any individual with a real LLM. Therefore, whenever there were fewer than 50 reference individuals with the same consensus nucleotide, the consensus nucleotide was not considered when calculating the empirical error rate (i.e., all individuals in the reference dataset were used).

The method described here has limited power to detect common variation, as the error profile estimated from the reference panel would actually represent the real mutation pattern. To solve this problem, when a position has a significantly higher error rate in most bins, the overall error rate estimated from all positions was used as the expected error rate to scan all positions (implemented in the Poisson method). Moreover, at all putative common variation positions, instead of using the empirical error rate in the subsequent steps, a given frequency threshold (or overall error rate) would be used as the empirical error rate, and this was flagged in the output file.
