# Supplementary material for: A new approach for detecting low-level mutations in next-generation sequence data
Source: Genome Biol. 2012 May 23;13(5):R34. doi: 10.1186/gb-2012-13-5-r34 (PMC3446287; doi:10.1186/gb-2012-13-5-r34)
Supplement: Additional file 3 — Supplemental tables. This file contains Tables S1, S2, S3, S5, and S6. [file gb-2012-13-5-r34-S3.PDF]

Additional data file 3

Supplemental table 1. Discovery rate and false positive rate under different sequencing depths with Poisson method

Minor allele frequency	Sequencing depth=200	Sequencing depth=500	Sequencing depth=1000	Sequencing depth=2000
2%	0	0	0.04	0.41
3%	0	0.03	0.34	0.96
4%	0	0.22	0.75	1
5%	0.02	0.38	0.95	1
6%	0.04	0.60	0.99	1
7%	0.10	0.80	1	1
8%	0.12	0.91	1	1
9%	0.18	0.96	1	1
10%	0.23	1	1	1
20%	0.54	1	1	1
False positive	0	0	0	0

Supplemental table 2. Discovery rate and false positive rate under different sequencing depths with Fisher Exact method

Minor allele frequency	Sequencing depth=200	Sequencing depth=500	Sequencing depth=1000	Sequencing depth=2000
2%	0	0	0.02	0.19
3%	0	0.02	0.18	0.88
4%	0	0.13	0.65	0.99
5%	0.02	0.26	0.92	1
6%	0	0.50	0.96	1
7%	0.04	0.66	1	1
8%	0.03	0.90	1	1
9%	0.23	0.91	1	1
10%	0.27	0.98	1	1
20%	0.98	1	1	1
False positive	0	0	0	0

Supplemental table 3. Discovery rate and false positive rate under different sequencing depths with Empirical method

Mutation level	Sequencing depth=200	Sequencing depth=500	Sequencing depth=1000	Sequencing depth=2000
2%	0	0.01	0.24	0.79
3%	0	0.15	0.64	1
4%	0	0.46	0.97	1
5%	0.03	0.68	0.99	1
6%	0.07	0.83	1	1
7%	0.15	0.96	1	1
8%	0.30	0.98	1	1
9%	0.41	0.99	1	1
10%	0.47	1	1	1
20%	0.99	1	1	1
False positive	0	0	0	0

Supplemental table 5. Empirical error rate at position 12857 on mitochondrial genome

Position on the read	Forward strand		Reverse strand	
	Read count	Error rate	Read count	Error rate
1:10	5000	0	7491	0.002
11:20	8184	0.001	4276	0.036
21:30	10628	0.002	2610	0.075
31:40	6387	0.002	1205	0.046
41:50	167	0	15	0.267
51:60	3374	0.001	167	0.581
61:70	2489	0.001	168	0.958
71:76	859	0.002	41	1
1:76	37088	0.002	15973	0.045

Supplemental table 6. Empirical error rate at position 295 on mitochondrial genome

	Forward strand		Reverse strand	
Position	Read count	Error rate	Read count	Error rate
1:10	144	0.007	6599	0.005
11:20	1140	0.003	5156	0.005
21:30	1804	0.004	1382	0.003
31:40	1429	0.003	345	0
41:50	193	0.005	179	0
51:60	292	0	432	0
61:70	363	0	217	0
71:76	15	0	45	0
1:76	5380	0.003	14355	0.005
